# Antiseptic Versus Asepsis

**Published:** 1918-10

**Authors:** 


### ANTISEPTICS VERSUS ASEPSIS

The value of the principle of antiseptics is called into question in military surgery, as it has been called into question in civilian surgery and for identical reasons. What are the adverse factors inherent in antiseptics?

*a*) The antiseptic injures the defending tissue.

*b*) Through the operation of the law of biologic adaptation through struggle and survival, antiseptics tend to breed a strain of bacteria of heightened degree of virulence.

*c*) To the extent the surgeon divides the responsibility of wound treatment with a chemical agent, to that extent he fails to develop clinically in judgment.

That all antiseptics injure living tissue needs no discussion; that the breeding of strains of high virulence may occur is equally established; and it is not at all unlikely that the virulent infection bred in hospitals, especially in the period of strong antiseptics, arose through natural selection. The clinician knows that after a few days or weeks of the same antiseptics, but little further benefit is noted. Change is necessary. This is probably owing to the adaptation of the bacteria to the antiseptic. Then, too, we have found in the civilian clinic that if wounds are kept free from pocketing, free from the accumulation of wound secretion, and a certain standard of care of the patient as well as of the wound, the presence or the absence of the antiseptic matters little. It is thought by some that the Carrel-Dakin method renders more possible than other methods the prevention of pooling in recessed wounds. Asepsia puts up to the surgeon the sole responsibility of wound care — scrupulous wound care; and when, owing to the nature of the wound or other circumstance, antiseptics do better, they are used.

The surgeon, who has an abiding faith in a specific for infection, deteriorates clinically. This has never been more obvious than in this war. We have only to recall each wave of hope and faith in a new certified antiseptic, and with it we recall a wave of depreciated surgical judgment. It takes pains, and thought, and application, and eternal vigilance, to do our best surgery, and we all welcome the let-down we unconsciously grant ourselves with each antiseptic wave. Every new antiseptic must not only make good on its own account, but it must also make up the surgical deficit in the surgical personnel for which it is indirectly responsible.

With the judgment shown by the British surgeons in this battle in selecting the cases for delayed treatment at the base and those for immediate treatment at the front, it would seem that antiseptics are likely to play a lesser role; for those cases operated at the front — such as abdomens, chests, thighs, and joints — required little or no antiseptics; and those that were without operative treatment until the 58 1/3 hour did no better with antiseptics than without, as shown by the tables. Thus, apparently, the field for antiseptics is contracting — but not disappearing. Attention is becoming more and more focussed on the leading role of sound surgery, of the natural defense inherent in the wound, of accurate judgment as to the cases to be evacuated or later operated and those to be immediately operated; attention is focussed on efficient transport system. Any one of these as a practical matter is more important in battle conditions than any one, even all the antiseptics thus far offered.

